# Forest floor plant diversity drives the use of mature spruce forests by European bison

**DOI:** 10.1002/ece3.7094

**Published:** 2020-12-16

**Authors:** Bogdan Jaroszewicz, Joanna Borysowicz, Olga Cholewińska

**Affiliations:** ^1^ Białowieża Geobotanical Station Faculty of Biology University of Warsaw Białowieża Poland

**Keywords:** Białowieża Forest, forb diversity, large herbivores, space use, temperate forest, ungulates

## Abstract

The distribution of large ungulates in space is in large extent driven by the availability of forage, which in temperate forests depends on light availability, and associated plant diversity and cover. We hypothesized that the increased number of GPS fixes of European bison (*Bison bonasus* L.) in usually avoided spruce forests was an effect of higher plant species richness and cover of the forest floor, which developed owing to increased light availability enhanced by spruce mortality. We carried out 80 forest floor plant surveys combined with tree measurement on plots chosen according to the number of GPS locations of GPS‐collared European bison. The mean plant species richness per plot was higher on intensively visited plots (IV) than rarely visited (RV) plots (30 ± 5.75 (*SD*) versus. 26 ± 6.19 (*SD*)). The frequency of 34 plant species was higher on IV plots, and they were mainly herbaceous species (32 species), while a significant part of 13 species with higher frequency on RV plots was woody plants (5 species). The species richness of forbs was higher on IV plots, while other functional groups of plants did not differ. Tree stem density on the IV plots was lower than on the RV plots (17.94 ± 6.73 (*SD*) versus 22.9 ± 7.67 (*SD*)), and the mean value of Ellenberg's ecological indicator for light availability for all forest floor plant species was higher on IV plots. European bison visiting mature spruce forests was driven by higher forest floor plant cover and species richness, and high share and species richness of forbs. The two latter features may be translated into higher quality and diversity of forage. In spite of morphological characteristics suggesting that European bison is a species of mixed (mosaic) habitats, it seems to be well adapted to thrive in diverse forests.

## Graphical abstract

Species composition, structure, and functioning of forest ecosystems are in large extent shaped by large herbivores. In our research, we studied visiting patterns of European bison (Bison bonasus L.) in mature spruce‐dominated stands, which are usually avoided by them. We found that high visiting rates were effect of higher forest floor plant cover and species richness and high share and species richness of forbs, which may be translated into higher quality and diversity of forage.

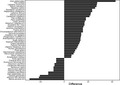

## 1 INTRODUCTION

Ungulates are important elements of forest ecosystems, shaping their structure, species composition, and functioning (Faison et al., 2016; Long et al., 2007). They influence forest vegetation via foraging, trampling, rooting, dispersing propagules, and many other behavioral activities. The intensity of pressure of ungulates on vegetation depends on the distribution and densities of their populations, which vary in time and space. In forest ecosystems, the distribution of ungulates depends on stand species composition, structure, forage distribution, predation risk, season of animal life cycle (e.g., mating, calving), plant phenology, human activities, and many other, often interacting, factors (Bubnicki et al., 2019; Krasińska & Krasiński, 2007; Theuerkauf & Rouys, 2008). Among the European ungulates, European bison (*Bison bonasus* L.) is considered less sensitive to at least two of these pressures: It does not have any predator, and therefore is free of the “predation risk,” and in contrast to other ungulates, it does not show strong aversion to sites visited by humans (Krasińska & Krasiński, 2007; Theuerkauf & Rouys, 2008; Zbyryt et al., 2018). However, if European bison–human interactions take place at short distance, they influence the behavior and distribution of this animal (Haidt et al., 2018). Therefore, the main factors influencing the distribution of European bison in forest ecosystems are those associated with behavior (e.g., females looking for safe place for calving, males roaming between herds during the rutting season; Krasińska & Krasiński, 2007) and water and forage quantity and quality: the need to optimize energy and nutrient intake (Bergman et al., 2001; Buxton et al., 1995).

Adequate forage quantity for a herbivore is a sufficient quantity of the preferred or tolerated plant species of acceptable quality (Xiao et al., 2020). Thus, forage quantity can be limited even when plant biomass is abundant if it does not meet species‐specific forage preferences and dietary requirements. Forage quality, that is, the nutritive value of forage, strongly depends on plant maturity and environmental conditions. It is highest in the early phenological stages, when plants develop many young, actively growing leaves, and decreases with plant maturity or close to the end of the vegetative season (Chapman et al., 2014). Both, forage quality and forage quantity strongly depend on light availability, which in forest ecosystems is controlled by canopy openness (Depauw et al., 2020; Lin et al., 2001; Poorter, 2019). However, light access affects the quantity and quality of available forage in a contrasting manner. Plant biomass increases with light availability, while the chemical quality of forage decreases (Poorter et al., 2019). High light intensity allows the faster growth of plants, but it is often associated with an increase in the C:N ratio in plant tissues (Hartley et al., 1997; Kuijper et al., 2013; Norton et al., 1991; Poorter et al., 2019), which decreases forage digestibility. As well as that high light availability increases the content of secondary metabolites in plant tissues (Bryant, 1987; Bryant et al., 1983, but see Poorter, 2019 and Reichardt et al., 1991), which decreases plant palatability and may affect foraging behavior and patch selection, depending on the animal's ability for detoxification of forage. Some plant species can be consumed only by specific herbivores adapted to neutralize these toxins, but are avoided by others (Koster, 2012; Vehviläinen & Koricheva, 2006). Finally, plant diversity, which in temperate climate increases with light availability, substantially increases the quality and quantity of plant biomass (Schaub et al., 2020).

Many European and North American ungulates of temperate forests (e.g., roe deer *Capreolus capreolus* L., red deer *Cervus elaphus* L., and white‐tailed deer *Odocoileus virginianus* Zimm.) prefer forest gaps as foraging patches (Campbell et al., 2004; Kuijper et al., 2013; Reimoser & Gossow, 1996; Welch et al., 1990). However, some studies did not find such a pattern for the same or other herbivores (Campbell et al., 2006; Johnson et al., 1995; Kuijper et al., 2013; Moser et al., 2008). Kuijper et al. (2013), studying a guild of ungulates composed of European bison, red deer, roe deer, wild boar (*Sus scrofa* L.), and European elk (*Alces alces* L.), reported higher density of tracks of all studied ungulate species and longer duration of animal visits in forest gaps than under closed forest. However, these differences were significant only for red deer, while other species, for example, European bison, did not show a significant preference toward forest gaps (Kuijper et al., 2013). However, Kowalczyk et al. (2019) reported that its diet structure reflects the preference of this large herbivore for open habitats. Thus, different ungulate species in the same conditions may show different preferences. Hobbs and Swift (1988) and Stewart et al. (2000) predicted that herbivores should prefer neither dark forests due to their low biomass availability nor big gaps open for the long term, where biomass is abundant but dominated by poorly digestible, mature vegetation. Therefore, large herbivores in forest ecosystems should select for patches under semi‐open canopy or in small, recently open gaps, where biomass availability is intermediate but with high quality of forage. However, this rule may not be universal for all large herbivores, as their patch selectivity depends on their evolutionary adaptations (e.g., grazers versus concentrate selectors) and plant selection (Raynor et al., 2016 and literature cited there).

**Figure 1:**
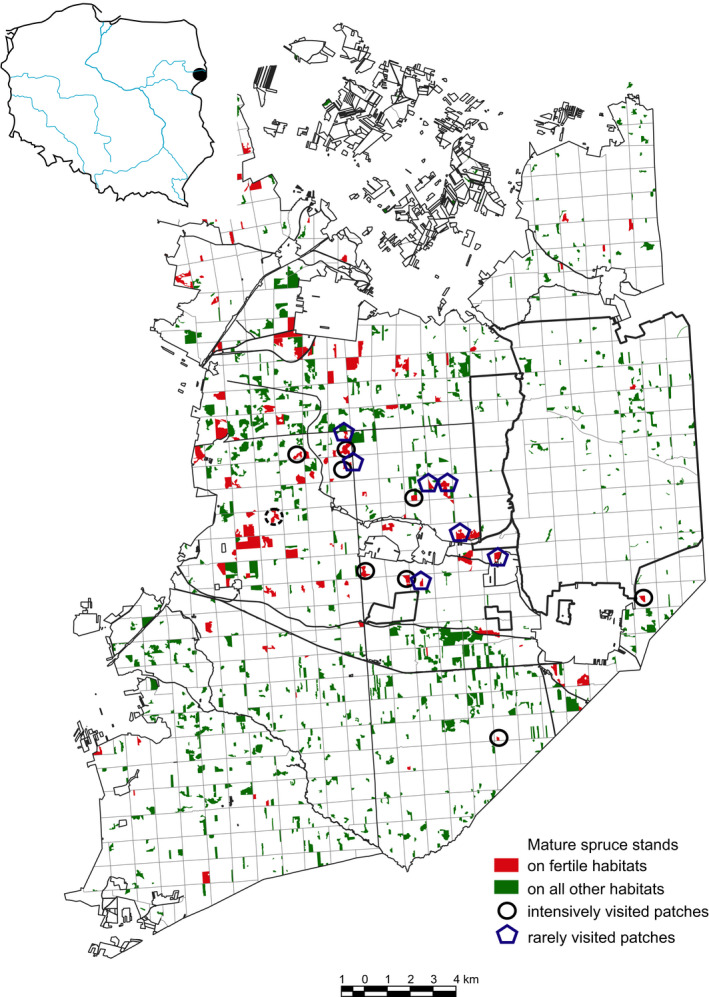
Distribution of mature spruce‐dominated stands (age > 70 years, spruce share > 50%) in the Polish part of Białowieża Primeval Forest (2012–2015). The circles and pentagons indicate forest sites chosen for vegetation surveys

The European bison, the largest terrestrial herbivore of Europe, went extinct in the wild at the beginning of the 20th century but has recently regained its range owing to long lasting protection, rebreeding, and reintroduction (Krasińska & Krasiński, 2007). Therefore, it is important to understand the habitat conditions, which are important for the distribution of this rare and still endangered species. Białowieża Primeval Forest (BPF), on the border between Poland and Belarus (Figure 1), has for centuries been a refuge site for this species (Samojlik et al., 2019), with the recent population on the Polish side of the border numbering 770 head (Raczyński, 2020). Contrary to the fact that the European bison is known nowadays mainly from forest ecosystems, several authors suggest that this species evolved in open or mixed mosaic habitats (Bocherens et al., 2015; Kerley et al., 2012), and in effect open areas should play an important role in its ecology. The foraging of European bison in forest gaps and open meadows was confirmed directly by field observations (Daleszczyk et al., 2007; Krasińska & Krasiński, 2007; Kuijper et al., 2013) and indirectly by revealing the high share of nonforest plant species remnants in its dung (Kowalczyk et al., 2019) and high share of nonforest plant species among endozoochorically dispersed seeds (Jaroszewicz et al., 2009). These sources unanimously suggest that species of open habitats play an important role in European bison diet and, consequently, forest gaps and clearings must play an important role in its foraging.

In the case of forest ecosystems, GPS fixes of European bison in BPF revealed that it prefers deciduous forests over coniferous, with the latter ones strongly avoided during the vegetative season (Jacob's selectivity index D = 0.36 versus D = −0.41, respectively; Szondelmejer, 2017). In spite of that, the European bison GPS fixes in the period 2012–2015 were concentrated in some mature (older than 70 years) stands of Norway spruce (*Picea abies* (L.) H. Karst) growing on fertile habitats. During the last decade, such stands were affected by a European spruce bark beetle (*Ips typographus* L.) outbreak (Gutowski & Jaroszewicz, 2016), the beginning of which overlapped with the start of the bison GPS‐tracking period. Due to the high shade casting ability of Norway spruce, which is one of the highest a the European tree species (Verheyen et al., 2012), spruce forests are usually dark (Evstigneev & Korotkova, 2019) and their forest floor and understorey poorly developed (Faliński, 1986). Therefore, we hypothesized that European bison, which normally avoid coniferous forests (Szondelmejer, 2017), were attracted there by an increase in forage availability and quality, associated with increased species richness and cover of the forest floor plants, resulting from the formation of forest gaps developed due to bark beetle‐caused spruce mortality. We carried out a field vegetation survey in spruce forest patches intensively visited and rarely visited by European bison to verify this hypothesis.

## 2 METHODS

### 2.1 Design and setting of the study

The study was carried out in Białowieża Primeval Forest (BPF)—one of the best preserved forest ecosystems on the European lowland (Jaroszewicz et al., 2019; Sabatini et al., 2018), which stretches over the border between Poland and Belarus (52.7°N, 23.9°E). This is approximately 1,500 km^2^ of hemiboreal, nemoral coniferous, and mixed broadleaved‐coniferous forests (vegetation types according to EEA, 2007), unique for its continuous tree cover and low degree of anthropogenic transformation for close to 12,000 years (Latałowa et al., 2016). In the 630 km^2^ western (Polish) part of the forest (Figure 1), where the research was conducted, habitats of deciduous and mixed deciduous forests prevail (oak‐lime‐hornbeam forests of the *Tilio‐Carpinetum* type), covering approximately 60% of the area (Faliński, 1986). Stands dominated by *Picea abies*, where our study was carried out, in 2011 covered 25.4% of the Polish part of BPF (Forest Data Bank https://www.bdl.lasy.gov.pl/portal/en). However, only a small portion of them were growing on potential habitats of mixed deciduous forests, not optimal for spruce, which made the tree vulnerable to European spruce bark beetle infestation (Gutowski & Jaroszewicz, 2016). Close to 27.5% of conifer dominated stands (13% of the total forest cover in BPF) during the years 2012–2018 were affected by the outbreak of the spruce bark beetle, which increased forest openness (Mikusiński et al., 2018).

Data from GPS‐locating of six European bison (three males and three females) from 2012–2015 were used to select stands for vegetation surveys (Table 1; Figure 1). These data were made available by the project “In situ conservation of European bison in Poland—northeastern part” executed in BPF by Warsaw University of Life Sciences (see acknowledgements for project details). Data on European bison distribution were gathered by GPS collars, which recorded the geographical coordinates of animals with 1‐hr frequency. To select study sites, we processed GPS fix data using QGIS software v2.18.2 (www.qgis.org). Only data from the vegetative period were considered (April‐October), because winter spatial distribution of the European bison in BPF is influenced by supplementary feeding (Krasińska & Krasiński, 2007), which makes environmental factors less relevant for their habitat preferences. The analyses were carried out on the background of forest inventory data (actual for 2010) obtained from the Forest Data Bank in 2016 (https://www.bdl.lasy.gov.pl/portal/en). In the first step, we selected forest stands fulfilling the species composition (>50% spruce), age (>70 years), and habitat (mixed deciduous mesic forest *Tilio‐Carpinetum*) criteria (Figure 1), which are the most vulnerable for bark beetle infestation (Grodzki, 2003). Stand was defined here as in forest management, as a patch of forest which differs from surrounding patches by: the way it was created, age, species composition, crown cover, density, technical quality of trees, habitat, etc., and which, thanks to its larger area, allows for a separate way of managing it (Szymański, 2000). In the second step, out of the set of stands fulfilling the selection criteria, those with the highest rate of European bison visiting (>5 GPS fixes during the period 2012–2015) were selected. The number of selected stands was limited due to intensive salvage logging carried out in BPF during the years 2016 and 2017 (Mikusiński et al., 2018). In effect, only 8 stands intensively visited by European bison (IV) were available for vegetation surveys, and the same number of the most similar stands in their closest neighborhood, fulfilling the same selection criteria but not visited or rarely visited (RV; <3 GPS fixes) by European bison, was selected as controls (Figure 1, Table 1).

**Table 1:** Number of GPS fixes of European bison (*Bison bonasus* L.) in mature Norway spruce (*Picea abies* (L.) Karst) stands in Białowieża Primeval Forest, NE Poland

Site code	Number of GPS fixes year^−1^				Area [ha]	Mean number of GPS fixes ha^−1^ year^−1^
	2012	2013	2014	2015		
**213D‐h**	**0**	**0**	**8**	**2**	**8.78**	**0.28**
215B‐b	0	1	0	2	7.1	0.11
**215D‐b**	**0**	**0**	**10**	**16**	**13.92**	**0.47**
247B‐d	0	1	0	0	2.57	0.10
**247B‐g**	**1**	**4**	**2**	**16**	**3.18**	**1.81**
**279C‐g**	**0**	**8**	**0**	**0**	**6.08**	**0.33**
280A‐d	1	0	0	0	3.73	0.07
280B‐c	1	0	0	0	7.3	0.03
304B‐c	0	0	0	0	1.75	0.00
339A‐h	0	0	0	0	2.36	0.00
**363A‐l**	**20**	**4**	**12**	**9**	**1.32**	**8.52**
368D‐h	0	0	0	0	9.41	0.00
**393B‐a**	**0**	**1**	**12**	**9**	**6.99**	**0.79**
394A‐c	0	0	0	1	3.6	0.07
**428C‐a**	**0**	**0**	**2**	**9**	**4.69**	**0.59**
**549C‐i**	**3**	**6**	**3**	**1**	**1.76**	**1.85**

Note

Intensively visited plots (IV) are indicated in bold.

In each IV stand, five European bison GPS locations were randomly chosen as geographical coordinates of vegetation survey plots in the field, resulting in a set of 40 plots. In the RV stands, the distribution of survey plots was randomly selected in the stand limits, also resulting in 40 plots (five plots in each of the eight studied stands).

### 2.2 Vegetation surveys

Vegetation surveys were carried out in May and June 2017—two years after the GPS‐tracking period ended. However, if the use of the forest by European bison was affected by bark beetle‐driven gap formation, then the development of light‐demanding vegetation should continue during the following years, and differences between the forest floor vegetation of the affected and unaffected forests should be even more pronounced. To avoid the observer effect on plot locations in the field, the central point of each research plot was determined with the use of a handheld GPS device (Garmin 60CSx), based on geographical coordinates extracted from a map created in QGIS software. We recorded all vascular plant species on 10 m × 10 m plots, and the cover of each species was assessed in the ten‐step Londo scale (Londo, 1976). In this study, three forest vegetation strata were considered, defined as: forest floor (all herbaceous plants and trees and bushes < 0.5 height), understory (all shrubs and trees with height 0.5 m < h < 6.0 m), and canopy (trees > 6.0 m height). The cover of each layer was visually estimated by observers as a percentage of the horizontal area covered by the vertical projection of tree crowns (canopy), bush crowns (understory), or herb plants (forest floor), respectively. Aside from vegetation surveys, we also measured the diameter at breast height (1.30 m) of all trees thicker than 7.0 cm on 400‐m^2^ circular plots (*r* = 11.28 m), whose central point was identical to the central point of the vegetation survey plot. Plant nomenclature in our paper follows Mirek et al. (2003).

### 2.3 Data analysis

Due to the variable size of the studied stands (from 1.32 to 13.92 ha), we calculated the mean number of European bison GPS locations per hectare per year as a proxy measure of intensity of stand use. The mean values of Ellenberg's ecological indicators and Shannon–Wiener indices were calculated, taking into account the percentage species cover as a weight. To determine the significance of differences in average value of indicators, we used the nonparametric Mann–Whitney *U* test and parametric Student's *t* test, depending on the data distribution. We calculated plant species frequency as a percentage of all plots where the species occurred (the number of plots where species was present/total number of IV or RV plots (respectively) × 100). Differences in species’ frequencies on IV plots and RV plots were assessed using the chi‐square test (significance level set at *p* = .05). To avoid the disproportional influence of rare species on the chi‐square test results, we considered only those species whose frequency was equal to or higher than 5%. Principal component analysis (PCA) was based on percentage species cover data. We chose the ordination method based on the gradient length of data distribution on the scatter plot. For data analysis, we used R 3.4.2 and Rstudio 1.1.383 software with *vegan* (Oksanen et al., 2019) and *ggplot2* (Wickham, 2016) packages.

## 3 RESULTS

The mean number of GPS locations of European bison in the intensively visited (IV) plots was significantly higher (Mann–Whitney *U* test: z = −3.3204, *p* = .0009) than in the rarely visited (RV) (1.83 ± 2.77 (*SD*) and 0.47 ± 0.46 (*SD*) ha^−1^ y^−1^; Table 1).

The Shannon–Wiener index of plant species diversity did not differ between the IV and RV plots (Sh = 2.67 ± 0.29 versus 2.60 ± 0.39, respectively; Welch two‐sample *t* test: *t* = 0.58, *df* = 76.95, *p* = .5574). The total species richness of RV plots was 7% higher than IV plots (138 species versus 129 species, respectively), while contrariwise, mean species richness per plot was higher on IV than RV plots (30 ± 5.75 (*SD*) versus 26 ± 6.19 (*SD*) species, respectively; Fig. B; Mann–Whitney *U* test: z = 3.113, *p* = .001) and the percentage plant cover of the forest floor was also higher on IV than RV plots (Mann–Whitney *U* test: z = 4.843 *p* < .001; Fig. B).

Most of the functional groups of plant species (graminoids, ferns, shrubs, and trees) did not differ by plant cover nor by mean species richness between the two studied plot categories. The only group whose mean species richness was higher on IV plots was forbs (Mann–Whitney *U* test; *p* = .0042).

**Figure 2:**
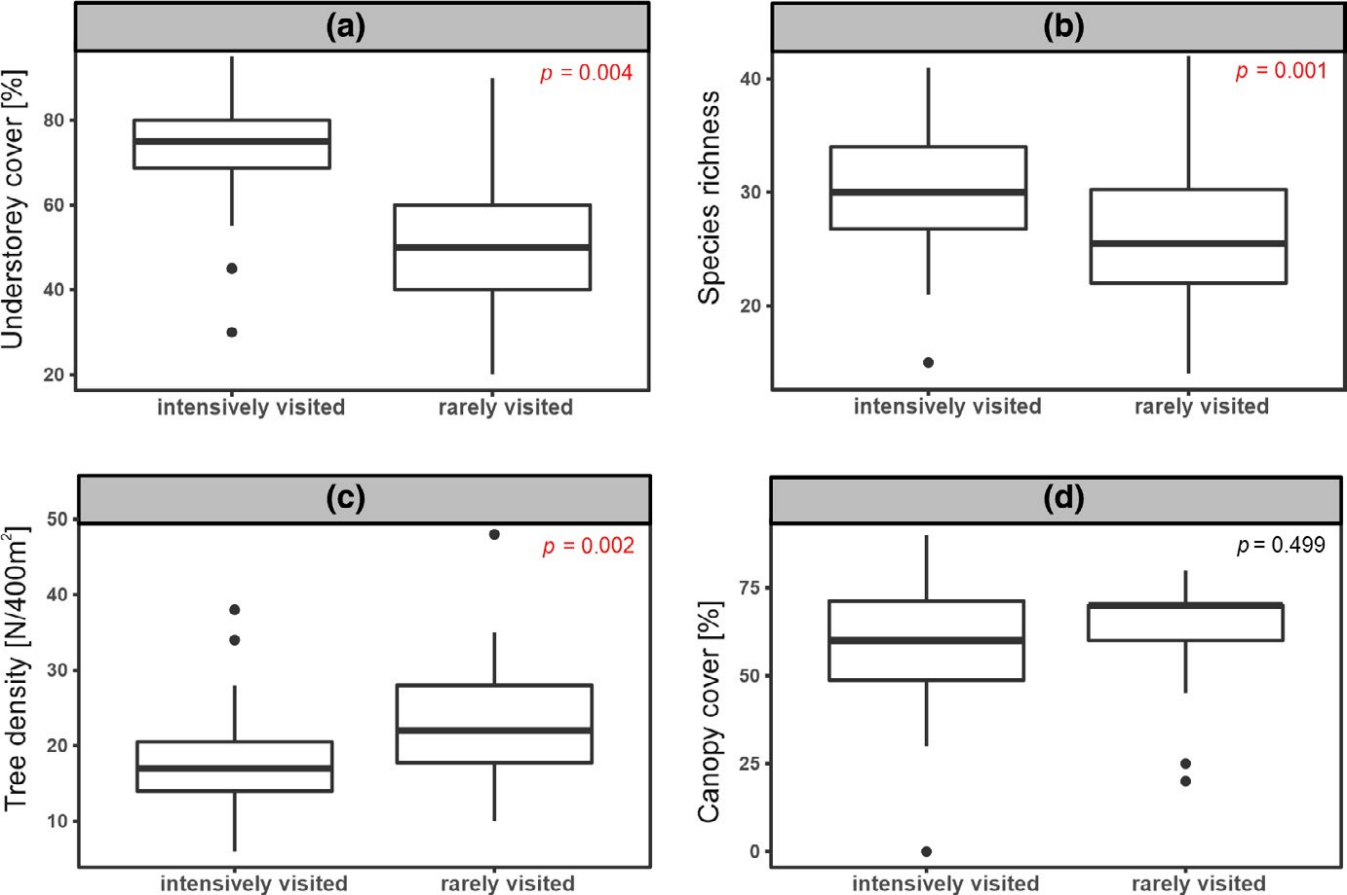
Differences in (a) forest floor plant cover (%), (b) forest floor species richness (mean number of species per 100‐m^2^ plot), (c) tree density (mean stem number per 400‐m^2^ plot) and (d) canopy cover (%) between intensively visited and rarely visited plots in spruce‐dominated stands in Białowieża Primeval Forest, NE Poland. The median is shown as a thick line in the middle of the boxplot

Tree stem density on the IV plots was lower than on the RV plots (17.94 ± 6.73 (*SD*) and 22.9 ± 7.67 (*SD*), respectively; Mann–Whitney *U* test: z = −3.026, *p* = .002; Figure 2). However, it did not affect tree canopy cover, which did not show significant differences between the plot categories (Mann–Whitney *U* test: z = −0.681, *p* = .4992; Figure 2).

**Figure 3:**
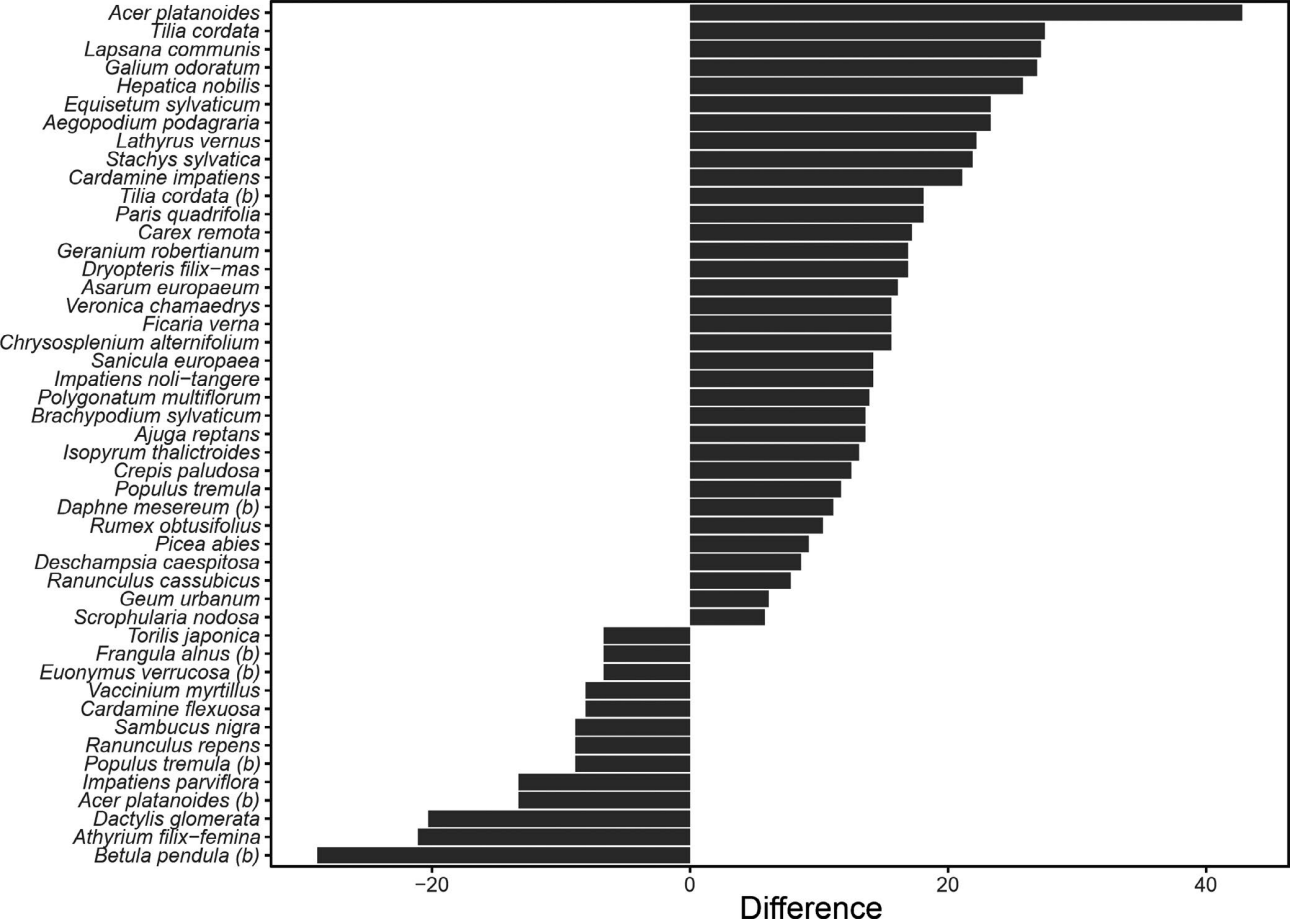
Significant differences (*p* < .05) in plant species frequency between intensively visited (IV) plots and rarely visited (RV) plots; (b) following the species name indicates trees and shrubs from the understory layer. Only plant species with frequency > 5% were considered

Out of 144 plant species recorded in total, 47 species revealed significant differences in frequency between the IV and RV plots (Figure 3; Appendix S1). The frequency of 34 species was higher on IV plots. These were mainly herbaceous species (32 species) with the mean value of Ellenberg's ecological indicator for light availability L = 3.92 ± 0.34 (*SD*). A significant part of species with higher frequency on RV plots (13 species) were trees and shrubs constituting the understory layer (5 species), with eight herbaceous species (Figure 3) characterized by the mean value of Ellenberg's ecological indicator for light availability higher than species with higher frequency on IV plots (5.47 ± 0.96 (*SD*) versus 3.92 ± 0.34 (*SD*); Mann–Whitney *U* test, z = −2.6, *p* = .009). However, the mean value of Ellenberg's ecological indicator for light availability for all forest floor plant species was higher on IV plots, with the significance of difference on the verge of the standard threshold of significance (L = 3.89 ± 0.6 (*SD*) versus L = 3.61 ± 0.63 (*SD*), respectively, Mann–Whitney U test: z = 1.895; *p* = .058). Other analyzed Ellenberg's ecological indicators of the forest floor plants (temperature, humidity, acidity, nutrients) did not differ between plot categories.

**Figure 4:**
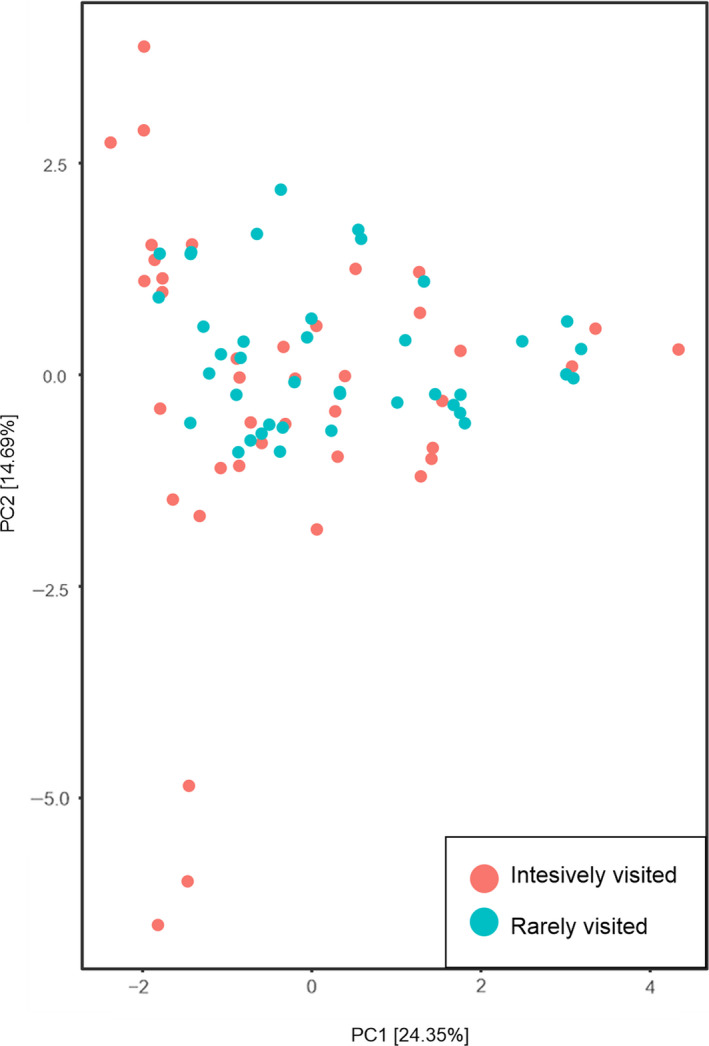
PCA scatter plot of species composition of the study plots intensively visited (IV) and rarely visited (RV) by European bison in mature spruce‐dominated stands in Białowieża Primeval Forest, NE Poland

Principle component analysis (PCA) did not reveal clustering of plots according to visiting intensity (Figure 4). The first two principle components of this analysis explained 39.04% of the total variance in plant species composition.

## 4 DISCUSSION

All studied stands were mature older than 70 years spruce‐dominated (>50% spruce in the stand) forests growing on the same type of fertile habitats (Forest Data Bank https://www.bdl.lasy.gov.pl/portal/en), which naturally should be occupied by mixed deciduous forests of the *Tilio‐Carpinetum* type. In effect, most of the vegetation properties (e.g., mean values of Ellenberg's plant ecological indicators, biodiversity measures, understorey species composition) did not differ between IV and RV sites. This was also confirmed by PCA analysis (Figure 4), which revealed that forest floor species composition of the IV and RV plots overlapped. Therefore, research plots represented equal habitat conditions, and observed differences in European bison visiting frequency had to result from other factors.

Contrary to our main hypothesis, that high intensity of use of the studied forest patches by European bison was caused by gap development, canopy cover of IV plots was quite high (over 60% on average) and did not differ from RV plots. Thus, the number of GPS locations of European bison in IV plots was not caused by the recent bark beetle infestation and consecutive canopy gap formation. However, IV plots in comparison with RV plots were occupied by plants typical for places with higher light availability, indicated by a higher mean value of Ellenberg's ecological indicator for light.

The higher number of GPS locations of European bison on the IV plots may be to some extent explained by the higher cover and species richness of forbs. Forbs increase forage attractiveness due to their high nutritive value. The nutritive value of forbs and woody plants is higher than grasses, because generally they contain more phosphorus, protein, and other nutrients (Bidgoli & Ranjbarfordoei, 2013; Holechek, 1984; Lee et al., 2018; Pearson et al., 1982). However, high forb abundance may also have an adverse effect on the distribution of some herbivores because plant secondary metabolites (phytotoxins) are often much more abundant in forbs and browse species than in grasses (Harborne, 1988). To ensure a nutritionally adequate diet, herbivores must overcome chemical plant defences and select plants that vary in nutrient value and toxicity in time and space (Foley & Moore, 2005; Laca & Demment, 1991; Provenza et al., 1990). This is a serious obstacle for selective and small bodied herbivores. However, the European bison is a large size generalist (Kowalczyk et al., 2019; Krasińska & Krasiński, 2007), consuming a wide array of over 450 plant species (Jaroszewicz and Pirożnikow, 2008) of varying quality. It may successfully cope with plant secondary metabolites owing to the large volume (>100 liters) of its ruminant stomach (Gill, 1967; Pytel, 1969). A large amount of plant biomass in the stomach buffers animal exposure to phytotoxins specific to a single plant species (Freeland & Janzen, 1974; Laycock et al., 1988) owing to its dilution in a diverse forage. Thus, the preference of European bison for plots with higher mean species richness, revealed in our study, may be an important part of this strategy. The higher the species richness of plants in a foraging patch, the higher the dilution effect should be if the toxic species is not dominant in the community. Another mechanism involved in the reduction of the phytotoxin loads of European bison is its foraging behavior: slow grazing associated with continuous slow movement (Krasińska & Krasiński, 2007). This foraging behavior increases the dilution effect by increasing the diversity of consumed forage. Wiggins et al. (2003) reported that captive animals challenged with high concentrations of plant secondary metabolites decrease the amount of single meals and expand their total feeding time, exactly as the European bison does (Krasińska & Krasiński, 2007). This behavior reduces instantaneous loads on animals’ detoxification systems.

The tree stem density on IV plots was lower than on RV plots, but the mean canopy cover did not differ between them. Nevertheless, forest floor plant cover, which may be treated as a proxy of plant biomass, was higher on IV plots, and the mean value of Ellenberg's ecological indicator for light availability of forest floor vegetation was also higher there. This enhanced the attractiveness of IV patches for European bison because they contained more biomass (higher plant cover), and their partial shading enhanced the palatability of forage by increasing its nutritious value and decreasing the content of plant secondary metabolites in comparison to better‐lit patches (Bryant et al., 1992; Harborne, 1991).

Our results suggest that European bison select places with higher food quality (expressed by higher species richness and higher share of forbs), and higher biomass to optimize foraging, which confirms our hypothesis that it is attracted by places with higher plant cover and species richness. This pattern was also well expressed in the selection by European bison of plots with high frequency of forbs and juvenile woody plants in the forest floor stratum. This result is in line with the feeding ecology of European bison, which is considered a mixed feeder, with a 67% winter and 97% summer share of herbaceous plants in its forage (Borowski & Kossak, 1972; Gębczyńska et al., 1991; Kowalczyk et al., 2019; Krasińska & Krasiński, 2007).

The European bison is capable of improving the quality of its habitats by increasing the species diversity of rare forbs in forests via extending the time of their increased openness, the dispersal of seeds of plants originating from nonforest habitats, and enhancing soil biological activity and fertility (Evstigneev et al., 2017; Ivanova et al., 2018; Jaroszewicz et al., 2009). The increase of heterogeneity, nutrient redistribution, changes in plant species composition, creation and maintenance of grasslands, increase in grassland productivity, and disturbance of woody vegetation were also reported for closely related American bison (*Bison bison* L.) from North America (Coppedge & Shaw, 1997; Gates et al., 2010; Hartnett et al., 1996; Knapp et al., 1999). Provenza et al. (2003) stated that browsing and grazing by mammalian herbivores can enhance or reduce plant chemical diversity and species diversity across landscapes. However, all such assessments of vegetation changes caused by both bison species (on both continents) were carried out in grasslands or on gaps in the forests, in more northern and more continental climates than BPF. Thus, it is difficult to clearly conclude how far differences observed in vegetation of the IV and RV plots were effects, and how far they were only a cause of high European bison visitation on the IV forest patches in BPF. It was reported that vegetation reacts with some lag to changes in environmental conditions (Wu et al., 2015). Most probably, more open patches attracted European bison similarly to other ungulates (Kuijper et al., 2013), whose grazing and browsing inhibited the development of young trees and shrubs, and increased forest floor species diversity owing to the reduction of competition between plants (by grazing), and owing to zoochoric seed dispersal (Jaroszewicz et al., 2009). Additionally, the diversity of forest floor vegetation increases owing to the deposition of feces, which enhances local soil fertility and biological activity (Evstigneev et al., 2017). European bison dung pats also enhance the establishment of plant species typical for fertile mixed deciduous forests, even in habitat conditions which are not optimal (Jaroszewicz & Pirożnikow, 2011).

## 5 CONCLUSIONS

The visiting of mature spruce forests by European bison was associated with higher forest floor plant cover and species richness, and the high share and species richness of forbs, in spite of moderate shade conditions. The high share and species richness of forbs may be translated into higher quality and diversity of forage. In spite of morphological adaptations (e.g., wide muzzle, hypsodont teeth) suggesting that from an evolutionary point of view European bison is a species of open or mixed (mosaic) habitats (Bocherens et al., 2015; Kerley et al., 2012; Mendoza & Palmqvist, 2008), it is well adapted to thrive in diverse forests (Kowalczyk et al., 2019). European bison may benefit from anthropogenic (silvicultural) and natural disturbances, which initiate the development of more open forest patches, where high forage diversity may develop, allowing a compromise between food quality and availability (Krebs & Davies, 1978; MacArthur & Pianka, 1966; Pulliam, 1974). A better understanding of these interactions is important in assessing the effect of European bison for habitat restoration goals (e.g., the preservation of open patches in forest ecosystems, preservation of light‐demanding forest plant species), as well as for conservation practices aimed at the reestablishment and maintenance of the European bison population in the future.

## CONFLICT OF INTEREST

The authors declare no conflicts of interest for this work.

## AUTHORS’ CONTRIBUTION

Bogdan Jaroszewicz: Conceptualization (Lead), Data curation (Equal), Formal analysis (Supporting), Investigation (Equal), Methodology (Lead), Writing—original draft (Lead), Writing‐review and editing (Equal); Joanna Borysowicz: Data curation (Supporting), Formal analysis (Supporting), Investigation (Equal), Writing—original draft (Supporting), Writing—review and editing (Equal); Olga Cholewińska: Conceptualization (Supporting), Data curation (Equal), Formal analysis (Lead), Investigation (Equal), Methodology (Supporting), Visualization (Lead), Writing—original draft (Equal), Writing—review and editing (Equal).

## Supporting information

Appendix S1

## Data Availability

The datasets used and analyzed during the current study are available at the Open Forest Data Repository: https://doi.org/10.48370/OFD/ANUMGX
