# Supplementary material for: Forest floor plant diversity drives the use of mature spruce forests by European bison
Source: Ecol Evol. 2020 Dec 16;11(1):636–47. doi: 10.1002/ece3.7094 (PMC7790634; doi:10.1002/ece3.7094)
Supplement: Supplementary file 1 — Appendix S1 [file ECE3-11-636-s001.docx]

**Appendix 1 to the article Jaroszewicz B., Borysowicz J., Cholewińska O. Forest floor plant diversity drives the use of mature spruce forests by European bison, Ecology and Evolution**

Table 1. Significant differences (p<0.05) in plant species percentage frequency between intensively visited (IV) and rarely visited (RV) plots; (b) following the species name indicates understory woody species (height 0.5 – 6.0 m). Only plant species with frequency >5% were considered.

| Species | Frequency | | ꭓ^2^ | p-value | difference |
| --- | --- | --- | --- | --- | --- |
|  | IV | RV |  |  |  |
| *Acer platanoides* | 85 | 42,2 | 14,401 | 0,000 | 42,8 |
| *Tilia cordata* | 47,5 | 20 | 11,204 | 0,001 | 27,5 |
| *Lapsana communis* | 45 | 17,8 | 11,781 | 0,001 | 27,2 |
| *Galium odoratum* | 82,5 | 55,6 | 5,240 | 0,022 | 26,9 |
| *Hepatica nobilis* | 52,5 | 26,7 | 8,405 | 0,004 | 25,8 |
| *Aegopodium podagraria* | 50 | 26,7 | 7,078 | 0,008 | 23,3 |
| *Equisetum sylvaticum* | 30 | 6,7 | 14,793 | 0,000 | 23,3 |
| *Lathyrus vernus* | 40 | 17,8 | 8,527 | 0,003 | 22,2 |
| *Stachys sylvatica* | 37,5 | 15,6 | 9,032 | 0,003 | 21,9 |
| *Cardamine impatiens* | 50 | 28,9 | 5,643 | 0,018 | 21,1 |
| *Paris quadrifolia* | 22,5 | 4,4 | 12,179 | 0,000 | 18,1 |
| *Tilia cordata (b)* | 42,5 | 24,4 | 4,897 | 0,027 | 18,1 |
| *Carex remota* | 35 | 17,8 | 5,603 | 0,018 | 17,2 |
| *Dryopteris filix-mas* | 32,5 | 15,6 | 5,938 | 0,015 | 16,9 |
| *Geranium robertianum* | 72,5 | 55,6 | 2,230 | 0,135 | 16,9 |
| *Asarum europaeum* | 25 | 8,9 | 7,646 | 0,006 | 16,1 |
| *Chrysosplenium alternifolium* | 20 | 4,4 | 9,974 | 0,002 | 15,6 |
| *Ficaria verna* | 20 | 4,4 | 9,974 | 0,002 | 15,6 |
| *Veronica chamaedrys* | 40 | 24,4 | 3,779 | 0,052 | 15,6 |
| *Impatiens noli-tangere* | 27,5 | 13,3 | 4,942 | 0,026 | 14,2 |
| *Sanicula europaea* | 27,5 | 13,3 | 4,942 | 0,026 | 14,2 |
| *Polygonatum multiflorum* | 25 | 11,1 | 5,352 | 0,021 | 13,9 |
| *Ajuga reptans* | 42,5 | 28,9 | 2,590 | 0,108 | 13,6 |
| *Brachypodium sylvaticum* | 22,5 | 8,9 | 5,890 | 0,015 | 13,6 |
| *Isopyrum thalictroides* | 17,5 | 4,4 | 7,836 | 0,005 | 13,1 |
| *Crepis paludosa* | 12,5 | 0 | 12,500 | 0,000 | 12,5 |
| *Populus tremula* | 25 | 13,3 | 3,574 | 0,059 | 11,7 |
| *Daphne mesereum (b)* | 20 | 8,9 | 4,263 | 0,039 | 11,1 |
| *Rumex obtusifolius* | 12,5 | 2,2 | 7,217 | 0,007 | 10,3 |
| *Picea abies* | 22,5 | 13,3 | 2,364 | 0,124 | 9,2 |
| *Deschampsia caespitosa* | 17,5 | 8,9 | 2,802 | 0,094 | 8,6 |
| *Ranunculus cassubicus* | 10 | 2,2 | 4,987 | 0,026 | 7,8 |
| *Geum urbanum* | 15 | 8,9 | 1,557 | 0,212 | 6,1 |
| *Scrophularia nodosa* | 12,5 | 6,7 | 1,752 | 0,186 | 5,8 |
| *Euonymus verrucosa (b)* | 0 | 6,7 | 6,700 | 0,010 | -6,7 |
| *Frangula alnus (b)* | 0 | 6,7 | 6,700 | 0,010 | -6,7 |
| *Torilis japonica* | 0 | 6,7 | 6,700 | 0,010 | -6,7 |
| *Cardamine flexuosa* | 7,5 | 15,6 | 2,840 | 0,092 | -8,1 |
| *Vaccinium myrtillus* | 7,5 | 15,6 | 2,840 | 0,092 | -8,1 |
| *Populus tremula (b)* | 0 | 8,9 | 8,900 | 0,003 | -8,9 |
| *Ranunculus repens* | 0 | 8,9 | 8,900 | 0,003 | -8,9 |
| *Sambucus nigra* | 0 | 8,9 | 8,900 | 0,003 | -8,9 |
| *Acer platanoides (b)* | 0 | 13,3 | 13,300 | 0,000 | -13,3 |
| *Impatiens parviflora* | 0 | 13,3 | 13,300 | 0,000 | -13,3 |
| *Dactylis glomerata* | 17,5 | 37,8 | 7,452 | 0,006 | -20,3 |
| *Athyrium filix-femina* | 30 | 51,1 | 5,490 | 0,019 | -21,1 |
| *Betula pendula (b)* | 0 | 28,9 | 28,900 | 0,000 | -28,9 |
